# Report of the Diseases of Edinburgh for June, 1809

**Published:** 1809-08

**Authors:** John Roberton


### *Report of the Diseases of Edinburgh for June,* 1809.

By John Roberton, M. D.

During the first third of the month the weather was generally soft, occasionally intermixed with some good days, and we had also during this period some falls of rain.

From this till near the beginning of the last third, the weather was very variable, with some hard gales of wind. It then improved remarkably, and we had some excellent days, when it again became cloudy and moist till the termination of the month.

The barometer, which was low at the beginning of the month, rose about the end of the first third of it; it then continued to vary till near the end of the month, when it again fell.

The thermometer varied considerably, being sometimes so low as 40, at other times as high as 70, of Fahrenheit's scale.

The diseases of this month have been neither numerous nor severe. They have principally been of a slight inflammatory sort; some cases of ophthalmia, catarrh, a sort of influenza, and fever.

The late alarming violence of inflammatory complaints has now abated, as scarcely any cases of great severity have occurred in practice since the commencement of the month.

Ophthalmia, though neither very prevalent nor of great severity, has appeared in some parts of the city. I have repeatedly observed, as I stated on a former occasion, that the severity of this disease is greatly increased when it occurs in persons of a scrofulous habit of body: indeed, on the present occasion, it is only in such that it is at all conspicuously severe. Where this habit of body prevails, the treatment must be very different from what is proper under other circumstances. Instead of any of the common cooling solutions, we must use those of greater activity. The holding the eye lids open and blowing into the eye a quantity of tincture of opium, or the introduction of a quantity of any kind of stimulating ointment between the eye lids, seems to be the only practice from which we are entitled to expect benefit. In very slight cases, simple bathing of the affected eyes with any kind of ardent spirit generally effects a cure; but, in those of very great severity, when vessels of considerable magnitude run over the ball of the eye, the only successful practice is to divide the vessels, and afterward employ such of the above applications as shall seem of greatest benefit.

Catarrh has prevailed rather extensively, and, in some instances, severely, especially when combined with cynanche tonsillaris, which has lately happened in various instances. Bleeding and purging, with the use of gargles, however, however, usually effected a perfect cure of these complaints.

A sort of influenza has lately made its appearance in this city. It too often appeared in combination with cynanche or catarrh, which rendered the cure of either of these affections much more tedious than they otherwise would have been. In some forms of this disease, it appeared, from the seemingly inflammatory state of the system that blood-letting might be of service; but the great debility which instantly followed this practice, prevented its being repeated or at all permitted. A disposition to faint remained with some patients for several days after such practice, and the debility was not in general recovered from in some instances even for two or three weeks. The most advisable practice seemed to be the administration of gentle saline purgative medicines, the application of blisters, and the use of a pill (of which I have frequently had occasion to take notice) composed of squills, g. ammoniac, myrrh, and a small proportion of opium. In all the cases which have come under my observation, I have found these means effect a perfect cure.

Fevers still continue to form a considerable part of the list of diseases. I think, of late, they seem to have prevailed with greater violence among children than in adults, and their consequences have in many instances been fatal.

Various are the forms which hysteric affections have assumed in different individuals, which have rendered the nature of this disease apparently complicated and perplexing. Two curious cases occurred to me sometime ago, both of which puzzled me very much; I shall briefly relate them both. The first was of a lady of about 40 years of age, stoutly made. She was suddenly seized with violent inflammation in the throat, and hoarseness, without any apparent cause: she applied to me, and I prescribed for her saline purgative medicines, and a gargle of diluted sulphuric acid; but, although she continued the use of these for several days, her complaint did not abate. From the character of the lady, and conversation with her husband on the subject, I had no reason to believe that the complaint was venereal, although in some respects it had something of that appearance. I directed a large blister to be applied to the throat, and the other prescriptions to be continued, but still her complaint was undiminished in severity. I was now much at a loss how I ought to proceed, when one day she told me that for the first time she that morning felt something rolling in her breast like

 a ball, a ball, and that it rose to her throat and had nearly suffocated her. I prescribed for this symptom, assafœtida and opium, and, to my utter astonishment, the inflammation in her throat was next day nearly gone, and the following day it was completely removed. She had frequent returns of this inflammation, though not nearly in such severity, and the assafœtida and opium uniformly removed them.

The other case was that of a lady about 30 years of age, of a delicate habit of body, who was evidently of a consumptive habit. For six or seven years she had been frequently troubled with a prickling sensation almost amounting to pain, in the soles of her feet, which, for several weeks together, often prevented her from putting her feet to the ground. Before I saw her, she had used sinapisms and blisters, electricity, and a variety of other external applications, from which she obtained momentary relief. She informed me, that during the intervals in which the prickling sensation left her, she was affected with other complaints, which, from her description, were evidently hysteric, and that all her complaints were much aggravated when her mind was in any way violently affected. I prescribed for her the volatile fœtid spirit, which completely removed these affections She died soon afterward of phthisis pulmonalis.

*No. 12, Princes Street.*

